# Comment impliquer le public et les patient(e)s dans la recherche en prévention primaire: Perspectives internationales et leçons tirées du réseau de recherche en prévention primaire des cancers CANCEPT

**DOI:** 10.17269/s41997-025-01135-0

**Published:** 2025-12-29

**Authors:** Pauline Oustric, Maria Claudia Addamiano, Niamh M. Redmond, Myriam Sève, Rita Di Giovanni, Claudia Diaz, Magali Ferrer Rigaud, Ana Millot, Carole Laffont, Myriam Dordonne, Charlotte Bauquier, Véronique Witkowski, Chloé Moulin, Pascale Journet, Harriet Rumgay, Melissa Gontero, Béatrice Fervers, Julien Biaudet

**Affiliations:** 1 Inserm U1296 Radiations: Défense, Santé, Environnement, Centre Léon Bérard, Lyon, France; 2 Département Prévention, Cancer Et Environnement, Centre Léon Bérard, Lyon, France; 3 Centre d’épidémiologie Et de Recherche en Santé Des Populations (CERPOP) - Équipe EQUITY, Université Toulouse III - Paul Sabatier, Inserm, Toulouse France; 4 Membre de GEM MIXTE Et Groupe de Recherche Participative COPRICA du Réseau CANCEPT, Lyon, France; 5 Promotion Santé Auvergne-Rhône-Alpes, Lyon, France; 6 Université de Rennes, EHESP, CNRS, InsermArènes—UMR 6051, RSMS—U 1309, Rennes, France; 7 Université Lumière Lyon 2, Inserm U1296, PôPS, Lyon France; 8 Centre International de Recherche Sur Le Cancer (CIRC), Lyon, France

**Keywords:** Patient and public involvement, Cancer primary prevention research, Public health, Stakeholders, Participatory research, Implication des patient(e)s et du public, Recherche en prévention primaire des cancers, Santé publique, Personnes concernées, Recherche participative

## Abstract

**Objectives:**

This article explores the methods and benefits of involving patients and the public in primary prevention (PP) cancer research. Through the experience of the CANCEPT PP research network, it aims to clarify practices and propose methodological guidelines for developing interventions tailored to the needs of populations.

**Methods:**

The Methodological Exchange Group GEM MIXTE, made up of 9 researchers and 9 co-researchers, met monthly (2023–2024, France) in order to define public and patient involvement in PP, take stock of participatory practices among CANCEPT members via a questionnaire (14 researchers, 8 institutions, 22 projects), and develop methodological guidelines in the form of a mind map. The participatory approaches were evaluated using the Public and Patient Engagement Evaluation Tool (PPEET) questionnaire.

**Results:**

GEM MIXTE has enabled us to clarify the methodology of participatory research in PP and to propose practical guidelines. This work highlights the importance of diversifying the profiles of the co-researchers to enhance the relevance of interventions, while emphasizing the role of the group coordinator in structuring and facilitating exchanges. The PPEET evaluation confirmed that the co-researchers were committed to the objectives, although socio-cultural diversity remains a challenge.

**Conclusion:**

This work proposes a methodological framework for integrating lived experience into research. The guidelines developed provide tools to inspire other networks to structure their approaches effectively, thereby strengthening citizen participation in public health.

## Résumé

**Objectifs:**

Cet article explore les modalités et bénéfices de l’implication des patient(e)s et du public dans la recherche en prévention primaire (PP) des cancers. À travers l’expérience du réseau CANCEPT, il vise à clarifier les pratiques et proposer des repères méthodologiques pour développer des interventions adaptées aux besoins des populations.

**Méthodes:**

Le Groupe d'Échange Méthodologique GEM MIXTE, composé de 9 chercheur(e)s et de 9 co-chercheures, s’est réuni mensuellement (2023–2024, France) afin de: livrer une définition de l’implication des patient(e)s et du public en PP, faire un état des lieux des pratiques participatives au sein des membres de CANCEPT via un questionnaire (14 chercheur(e)s, 8 institutions, 22 projets), élaborer des repères méthodologiques sous forme de carte mentale. L’évaluation des démarches participatives a été réalisée à l’aide du questionnaire Public and Patient Engagement Evaluation Tool (PPEET).

**Résultats:**

Le GEM MIXTE a permis de clarifier le cadre méthodologique de la recherche participative en PP et de proposer des repères pratiques. Ces travaux mettent en évidence l’importance de diversifier les profils des co-chercheur(e)s pour enrichir la pertinence des interventions, tout en soulignant le rôle du coordinateur pour structurer et animer les échanges. L’évaluation PPEET a confirmé l’adhésion des co-chercheurs aux objectifs, bien que la diversité socioculturelle reste un défi à relever.

**Conclusion:**

Ce travail propose un cadre méthodologique pour intégrer les savoirs expérientiels dans la recherche. Les repères élaborés offrent des outils pour inspirer d’autres réseaux à structurer efficacement leurs démarches, renforçant ainsi la participation citoyenne en santé publique.

## Contexte

La prévention primaire (PP) est un levier essentiel pour réduire l'incidence des cancers, 30% d'entre eux étant associés à des facteurs de risque nutritionnels et environnementaux accessibles aux actions de prévention, tels que le tabac, l'alcool, une alimentation déséquilibrée, le surpoids, la sédentarité ou les polluants (INCA, 2018). Pourtant, ces connaissances se traduisent rarement en actions efficaces. La communication et l’information, bien qu'importantes, ne suffisent pas à induire des changements de comportement durables, et les inégalités sociales face au risque de cancer persistent (Vaccarella et al., 2019). Impliquer activement les personnes concernées apparaît crucial pour surmonter ces limites et renforcer l’impact des actions de prévention. Dans cet article, l’ensemble des membres du public concerné par les enjeux de PP des cancers (patient(e)s, aidant(e)s, usager(e)s, etc.) sera désigné par le terme “personnes concernées”, le rationnel pour ce choix sera décrit dans la partie résultats.

Pour répondre à ces défis, notre réseau de recherche en PP CANCEPT, financé par l’Institut National du Cancer (INCa), réunit chercheur(e)s, professionnel(le)s de santé, praticien(ne)s et personnes concernées pour intégrer les savoirs scientifiques et expérientiels dans la PP des cancers. En s’appuyant sur la recherche interdisciplinaire, l’expertise de terrain et les connaissances des parties prenantes, CANCEPT vise à développer des interventions innovantes en PP. De nombreuses études et lignes directrices (Fondation Sciences Citoyennes, 2013; Hoddinott et al., 2018; National Institute for Health and Care Research (NIHR), (2024); OMS, 1999) démontrent que la recherche en santé est plus efficace lorsqu’elle implique les personnes directement concernées: la participation des personnes concernées apporte une valeur ajoutée en générant des questions de recherche pertinentes, en clarifiant les objectifs, en optimisant la conception des interventions, en améliorant le recrutement et la rétention des participant(e)s, et en renforçant la communication et la diffusion des résultats.

La recherche participative est définie comme « une forme de production de savoir qui implique une participation active des personnes concernées. Dans la pratique, c’est une science qui essaie de prendre en compte l’expérience des individus touchés – patient(e)s, aidant(e)s, soignant(e)s – pour produire un savoir complémentaire du savoir académique» (INSERM, 2023). Cette implication peut être à différents niveaux décrits par « l'échelle de participation» (Arnstein, 1969; Lochmüller et al., 2018) et à différentes étapes du projet de recherche (NIHR Bristol BRC, 2023). On retrouve ainsi différentes dénominations comme la recherche communautaire qui exige un partage des responsabilités entre les parties prenantes pour favoriser la mise en pratique des résultats de la recherche et, à terme, améliorer la qualité de vie de la population (Israel et al., 1998). Cela est particulièrement pertinent dans le contexte de la prévention du cancer (Larkey et al., 2009). Dans cet article, le terme recherche participative fera donc référence à toutes les formes d’implication de la consultation au partenariat.

Bien que les avantages de l’implication des personnes concernées dans la recherche soient ainsi largement reconnus, son application spécifique à la PP reste limitée (Bergin et al., 2023; Clet et al., 2024; Morisset et al., 2009). La PP, qui cible le grand public pour prévenir l’apparition des maladies, nécessite d’adapter les cadres existants de la recherche participative. En effet, des questions clés demeurent: qui impliquer, et comment définir et intégrer le savoir expérientiel des personnes concernées dans ce contexte? Un consensus sur ces enjeux est essentiel pour structurer l’implication des personnes concernées, et, *in fine*, maximiser l’efficacité des interventions de prévention.

**Fig. 1:**
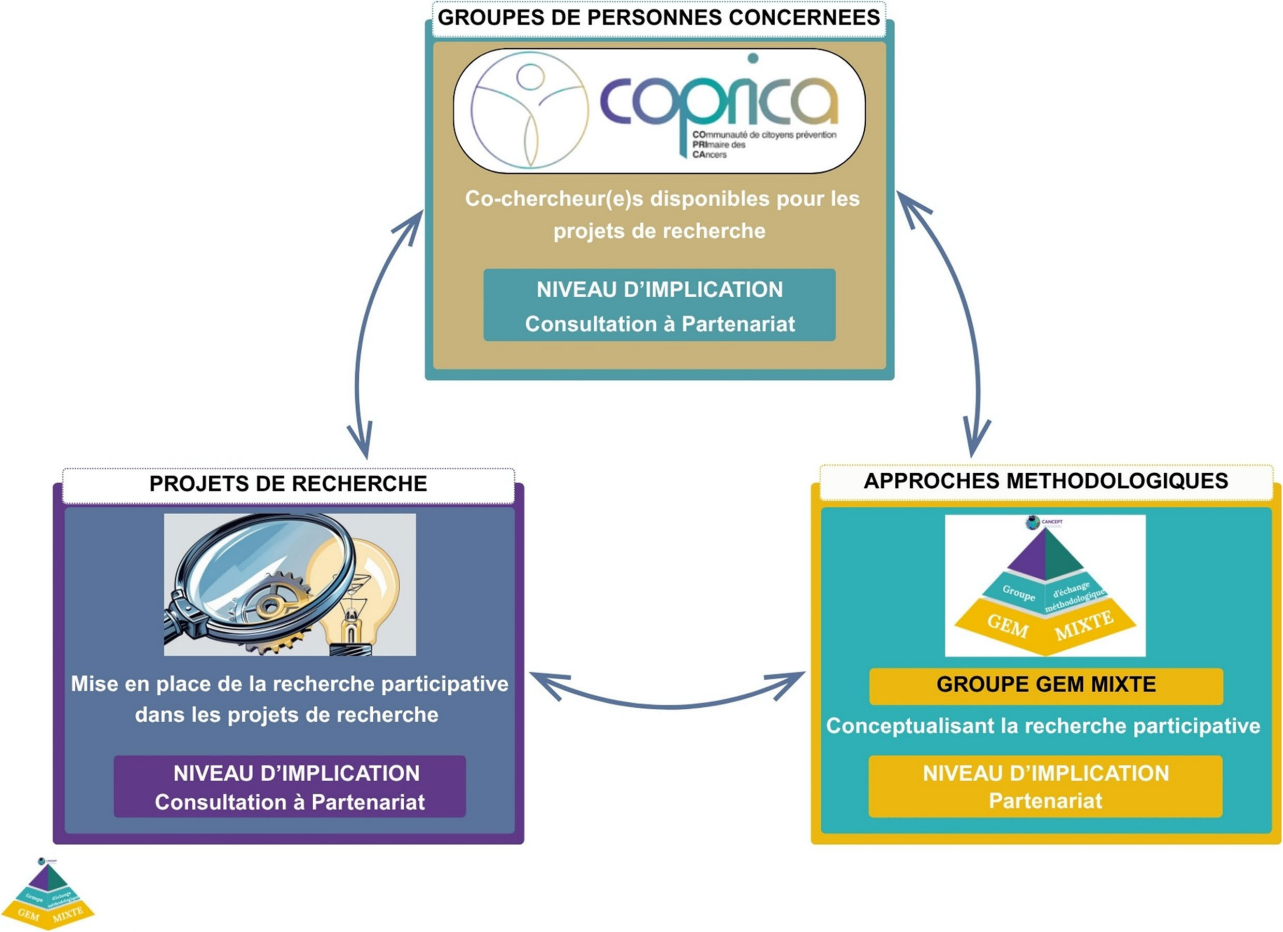
Recherche participative au sein de CANCEPT: 3 axes structurants, NIVEAU D'IMPLICATION: Information- Consultation-Collaboration-Partenariat selon le Model de Monréal, COPRICA: Communauté de Citoyens pour la Prévention Primaire des Cancers-GEM: GEM: Groip d'Echange Méthodologique-GEM MIXTE: Groupe d'Echange Méthodologique MIXTE-CANCEPT: Réseau de Recherche Transdisciplinaire sur la Prévention Primaire du Cancer, la Nutrition et l'Environnement (Cancer Primary Preventon Transdisciplinary Nutrition and Environnement Research Network)

L'objectif de cet article est de proposer un cadre méthodologique pour faciliter l'implication des personnes concernées dans le contexte spécifique de la recherche en PP, en s’appuyant sur les leçons tirées du réseau de recherche en PP CANCEPT. Cet article présente le travail du groupe participatif GEM MIXTE, qui a défini l’implication des personnes concernées en PP, puis évalué les pratiques participatives au sein de CANCEPT, pour élaborer des repères méthodologiques. Nous espérons inspirer d’autres réseaux, favoriser la collaboration entre chercheur(e)s et personnes concernées, et contribuer à la conception d’interventions de PP plus efficaces et participatives. Les termes propres au réseau CANCEPT et à ses différents axes sont définis dans la Fig. 1.

## Méthode

### Intégration de la recherche participative au sein du réseau CANCEPT

Au sein du réseau CANCEPT, la recherche participative a été développée selon trois axes structurants (Fig. 1) qui se renforcent mutuellement. Un groupe ressource de personnes concernées pour favoriser la recherche participative, COPRICA, a été planifié et créé dès le lancement du réseau en 2022. COPRICA, coordonné par une Community Manager, a recruté à ce jour 14 membres intéressés par la recherche. Ces personnes concernées peuvent intervenir à tous les niveaux d’implication et étapes des projets de recherche CANCEPT et la méthodologie de ce groupe est en cours de rédaction pour publication. Ensuite, un groupe d’échange méthodologique (GEM) réunissant des chercheur(e)s de disciplines variées de chaque institution de CANCEPT a été créé pour établir des méthodes et un cadre conceptuel commun. L'un de ses objectifs était de faire progresser la méthodologie de la recherche participative, dans le domaine de la PP du cancer. Le dernier axe correspond à la mise en place de la recherche participative dans les projets de recherche du réseau. À ce jour, 10 projets de recherche participative ont été développés au sein de CANCEPT avec différents niveaux de participation allant de la consultation au partenariat.

Ce schéma (Fig. 1) montre que la recherche participative dans CANCEPT repose sur trois piliers qui se renforcent mutuellement. Le groupe de personnes concernées COPRICA fournit des co-chercheur(e)s, le GEM MIXTE pose le cadre méthodologique, et les projets permettent une mise en pratique concrète de cette approche.

### Recrutement des membres de GEM MIXTE

Le groupe transdisciplinaire GEM, initialement formé par 9 chercheur(e)s venant des 8 institutions membres du réseau, a évolué pour intégrer des personnes concernées issues du groupe participatif COPRICA, devenant ainsi le GEM MIXTE. 9 personnes concernées, âgées de plus de 40 ans et issues de milieux sociaux divers, ont été recrutées via une présentation du projet au groupe COPRICA, suivie d’une invitation par email pour rejoindre GEM MIXTE. Ce projet visait à impliquer des personnes concernées afin de réfléchir avec elles aux savoirs expérientiels spécifiques à la PP. Le recrutement pour le groupe GEM MIXTE s’est fait au sein du groupe COPRICA, constitué dans le cadre du réseau CANCEPT.

### Réunions mensuelles de GEM MIXTE et établissement d’un plan d’action

Le GEM MIXTE a opté pour des ateliers mensuels de 1h30, une durée jugée adéquate par le groupe pour équilibrer un travail partenarial approfondi avec le respect des emplois du temps de chacun. Ces réunions se sont déroulées en visioconférence, les participant(e)s co-chercheures comme chercheur(e)s étant localisés dans toute la France. Une chercheure coordonne ces réunions afin d’assurer la création d’une culture commune, faciliter le lien entre les membres du groupe, assurer la démarche partenariale mais aussi les aspects pratiques rédactionnels. Lors de la première réunion, les objectifs du groupe visant à proposer un cadre méthodologique pour favoriser la recherche participative en PP au sein de CANCEPT ont été proposés, discutés et approuvés. Une charte d'engagement mutuel (ANNEXE 1), basée sur des lignes directrices de bonnes pratiques pour la recherche participative (NIHR, 2022) a été proposée et validée pour définir clairement les rôles et les niveaux d’implication. Cette charte met l'accent sur l’intégration des personnes concernées co-chercheures comme des membres à part entière de l’équipe de recherche. Conformément aux règles de COPRICA, une valorisation financière sous la forme de « chèque cadeau de 40 euros» a été proposée, approuvée et ainsi remise aux co-chercheures après chaque réunion. En complément de la valorisation financière, les co-chercheures bénéficient également d’une reconnaissance scientifique en tant que co-auteures. Chercheur(e)s et co-chercheures ont été invité(e)s à suivre la formation FORCES (FORmation Coproduction En Santé) (Unisanté, 2022) sur la recherche participative et l’éthique. Tous ont contribué à la rédaction de ce manuscrit, individuellement ou en sous-groupes selon les besoins.

Le plan d'action du groupe GEM MIXTE visait à répondre à la question « Comment faciliter l’implication du public et des patient(e)s dans la PP du cancer? ». Ce plan a été défini collectivement en 3 étapes:

1. Définir l’implication des personnes concernées et leurs savoirs expérientiels dans le cadre de la PP.
2. Dresser un état des lieux de l’implication des personnes concernées au sein du réseau CANCEPT.
3. Proposer des recommandations facilitant la recherche participative en PP.

Pour l’étape 1, l'enjeu principal a été de construire un cadre commun, autour de définitions claires. Il a été essentiel de préciser les spécificités de la recherche participative en PP, ainsi que de définir le rôle et l’implication des parties prenantes: Qui participe? Est-ce que tou(te)s les citoyen(ne)s peuvent participer à la recherche en PP des cancers? Qu'entend-on par « citoyen(ne)» et quels sont les « savoirs» que ce dernier(es) possède? Peut-on parler de savoirs expérientiels du citoyen?

Pour l’étape 2, un questionnaire a été co-conçu par le groupe GEM MIXTE, pour dresser un état des lieux et explorer les intérêts ainsi que les freins à la recherche participative selon les chercheur(e)s porteurs de projet(s) de CANCEPT. Ce questionnaire a repris les étapes de la recherche participative du NIHR et les niveaux d’implications du modèle de Montreal. L'annexe 2 détaille le questionnaire. La version finale a été soumise à une phase test auprès de chercheur(e)s afin d'en vérifier la clarté puis mise en ligne (Framaforms.org) et envoyée à tous les chercheur(e)s du réseau CANCEPT. Les réponses ont été analysées par le groupe GEM MIXTE afin d’en proposer une synthèse.

Pour l’étape 3, le groupe a mené un brainstorming collectif basé sur la littérature scientifique. Cette démarche a permis d’identifier des questions clés pour structurer les projets participatifs: évaluer la pertinence de l’implication des personnes concernées, définir leur rôle, élaborer une stratégie de recrutement et de valorisation, instaurer une culture commune, encourager une dynamique de groupe et développer des modalités d’évaluation.

Les observations et les retours d'expérience des ateliers ont été systématiquement enregistrés et des comptes-rendus ont été rédigés par la coordinatrice et validés par tous. Tous les membres ont eu accès aux comptes-rendus et travaux sur un Drive partagé et ont eu la possibilité d'ajouter des réflexions supplémentaires.

L’implication des personnes concernées a été évaluée à l'aide du questionnaire validé Public and Patient Engagement Evaluation Tool (PPEET) (Abelson and Université McMaster, 2018). Cet outil sert à identifier les forces et les axes d’amélioration des démarches participatives.

## Résultats

### Définir l'implication des personnes concernées en PP des cancers

#### La recherche participative en PP des cancers

Ce travail partenarial, centré sur la PP a nécessité de préciser ses spécificités par rapport aux préventions secondaires et tertiaires.

La PP, en tant qu’approche globale, vise à agir sur les déterminants sociaux de la santé, comme les comportements individuels, mais aussi l'environnement et les politiques publiques. L’expérience montre que les connaissances scientifiques sont nécessaires mais non suffisantes pour agir sur l’ensemble des facteurs de risque ainsi que sur la promotion des facteurs protecteurs (Syme, 2007). En croisant les différentes compréhensions de la définition de la PP, les discussions ont fait émerger l’idée que l’objet de la recherche porte davantage sur les facteurs de risque des maladies que sur les maladies elles-mêmes. La recherche participative en PP permet de travailler spécifiquement en amont sur ces facteurs, et notamment sur les freins aux pratiques préventives liés aux habitudes de vie, ainsi que sur les contraintes, opportunités ou influences d’un contexte spécifique (géographique, socio-économique ou politique).

#### Caractéristiques des participants en recherche participative en PP des cancers

Contrairement à la recherche sur une pathologie, qui requiert une expérience directe de la maladie, la recherche en PP des cancers concerne l'ensemble de la population. Le Groupe de Réflexion avec les Associations de Malades (GRAM) de l’INSERM utilise le terme « parties prenantes» (Docagne et al., 2023) où les usager(e)s, patient(e)s, aidant(e)s sont considérés au même titre que les professionnel(le)s de santé. Le groupe GEM MIXTE, a souligné l’importance de différencier dans ces parties prenantes celles qui proviennent d’un savoir issu de l’expérience du citoyen(ne) et celle des professionnel(le)s. De surcroît, le groupe a préféré au terme de “citoyen(ne)s” l’expression de “personnes concernées par la PP”. En effet, le premier terme exclut ceux qui ne jouissent pas de ce statut, par exemple, à cause de leur situation migratoire. Les « personnes concernées», telles que définies par le groupe GEM MIXTE, sont donc des parties prenantes de la recherche participative soit à titre individuel, soit en tant que représentant d’une communauté (associations des usagers, de consommateurs, de prévention, ligues, …).

Toutefois, une vigilance aux éventuels conflits d’intérêts des collectifs participants est nécessaire, par exemple, pour des associations qui reçoivent des financements des industries du tabac ou de l’alcool, situation qui met en cause la neutralité de leurs propos.

### Les savoirs expérientiels en PP des cancers

Selon Gardien (2017) les savoirs expérientiels, conceptualisés dans les années 1970, désignent des connaissances issues de l’expérience personnelle, en opposition aux savoirs théoriques. Ils se construisent à travers l’interaction entre l’individu et son environnement, influencés par les normes sociales et culturelles. Souvent associés à la maladie, ils englobent également des expériences du quotidien. Ces savoirs sont transmis de manière inégale selon les contextes sociaux.

Les savoirs expérientiels peuvent être représentés par des individus ou des collectifs (Groupes d’entraide mutuels, associations de malades, associations des usagers, ligues) correspondant à une expérience de vie ciblée. Par exemple, pour ses travaux sur le lobbying de l'industrie de l'alcool et de l'industrie du tabac, l’EHESP collabore avec différentes associations pour co-construire des programmes de recherche. Ces structures constituent aussi un relais en contribuant au recrutement de participant(e)s.

Concernant la PP, chacun dispose de savoirs expérientiels liés à son vécu de la santé et de ce qui la détermine. Ces savoirs sont essentiels pour comprendre les comportements et identifier les freins et leviers. Leur prise en compte offre l’opportunité d’élaborer des stratégies mieux adaptées pour promouvoir la santé. Dans le cadre du GEM MIXTE, les discussions nous ont amenés à définir plus précisément les savoirs expérientiels des personnes concernées à valoriser en PP:

- Les connaissances liées à l'expérience de la vie quotidienne, et des facteurs de risques,
- Le savoir être avec l'envie de partager ses représentations, expériences,
- Le savoir-faire pour analyser sa propre expérience et en tirer des enseignements utiles.

Les différentes compétences des participant(e)s, peuvent être une plus-value pour les projets de recherche et les résultats qui en découlent, à la condition d’être recueillies de manière méthodique en garantissant la libre expression des parties prenantes. Idéalement, la recherche participative devrait intégrer une diversité de profils (statut socio-économique, genre, âge, culture, milieux de vie …). Précisons que nous employons volontairement le terme diversité et non pas représentativité, celui-ci renvoyant au champ statistique auquel nous ne nous référons pas. Certains projets nécessitent de cibler des personnes aux caractéristiques spécifiques (ex: riverains d’un quartier) dès leur conception, mais inclure d’autres profils peut être tout aussi pertinent à d’autres étapes du projet.

Enfin, les échanges du groupe GEM MIXTE ont fait émerger l’idée que les savoirs expérientiels se définissent également en fonction des projets de recherche et de leur problématique. Les savoirs expérientiels se construisent dans la relation entre la personne concernée et l’objet étudié. Par exemple, un habitant d’un territoire exposé à des polluants industriels peut développer des connaissances liées à cette exposition qui, dans le cadre d’un projet de recherche, deviennent des savoirs expérientiels. Les connaissances tacites et les stratégies individuelles se formalisent grâce à des expériences sociales comme les discussions et l’engagement collectif, transformant ainsi ces connaissances en savoirs expérientiels.

Cette réflexion déplace le questionnement initial: plutôt que de débattre de l’existence de "savoirs expérientiels" des personnes concernées en PP, à l’instar de ceux des patient(e)s en santé, il s’agit d’explorer comment ces savoirs se construisent, dans le monde social, avant la recherche. Cela soulève la question du profil des individus à impliquer: une personne concernée ayant un rapport peu réflexif à l’objet étudié, dont les connaissances tacites seront formalisées en savoirs expérientiels au cours du projet, ou bien, à l’inverse, une personne concernée ayant un fort rapport réflexif à l’objet (comme une personne militante) dotée de savoirs expérientiels déjà constitués.

### Dresser un état des lieux de l’implication des personnes concernées au sein du réseau CANCEPT avec la co-construction et l'analyse d'un questionnaire adressé aux chercheur(e)s du réseau CANCEPT

Après avoir établi un langage commun, un questionnaire a été conçu pour recenser au sein du réseau CANCEPT les démarches participatives existantes et identifier les besoins, freins et leviers pour les renforcer. Le réseau CANCEPT a cartographié 33 projets de recherche en PP portés par ses équipes membres entre 2022 et 2024. 22 de ces projets ont été considérés comme des projets de recherche participative. 14 chercheur(e)s couvrant bien les 8 institutions constituant le réseau CANCEPT ont répondu à notre questionnaire par rapport à leur(s) projet(s) porté(s)**.** Une synthèse des résultats est reportée dans le Tableau 1:

**Tableau 1:** Etat des lieux de la recherche participative au sein du réseau CANCEPT

Réponses	14 Chercheur(e)s (représentants 8 institutions du réseau CANCEPT) répondant pour un ou plusieurs projets de recherche	
Mise en œuvre de la Recherche Participative	Oui: 10 réponses	Non: 4 réponses
Pourquoi?	Répondre aux besoins et aux inquiétudes des personnes concernées dans des cas précis	Méconnaissance du domaine et de la méthode
	Bénéficier de leurs propres savoirs et expériences spécifiques	Manque d’expertise et de connaissances
	Adapter l’intervention au plus grand nombre	Temporalité du projet ne permet pas d’inclure des co-chercheur(e)s
	Développer de nouvelles questions de recherche	Temps, démarches participatives plus chronophages
	Avoir un avis sur les travaux effectués et sur l’utilité des outils développés. – Valeur ajoutée scientifique	Thème de recherche peu en lien avec des associations déjà implantées ou “L’enjeu local du projet ne rendait pas pertinente la sollicitation de COPRICA à ce stade”
	Aller plus loin que ce que se seraient "permis" une équipe composée de chercheur(e)s uniquement	Besoin d’avoir un soutien méthodologique et d’avoir accès à des exemples de projets participatifs comme modèle
Étapes d’implication (NIHR Bristol BRC, 2023)	Les personnes concernées sont impliquées dans toutes les étapes d’un projet de recherche (ex: priorisation du sujet, conception, appel à projet, recrutement, analyse, valorisation, implémentation, évaluation …)	
	Le recrutement et l’analyse des données ainsi que la diffusion des résultats sont des étapes d’implication des co-chercheur(e)s souvent représentées	
	Les co-chercheur(e)s interviennent majoritairement dans l’étape de conception de projets mais peu dans l’évaluation ou les appels à projets	
Niveau de participation (Pomey et al., 2015)	Selon le modèle de Montréal, les approches participatives mises en œuvre par les répondants se répartissent ainsi: consultation (4 répondants), collaboration (2 répondants) et partenariat (3 répondants)	
	Le niveau « information» apparait également mais reste minoritaire (1 répondant)	
Recrutement	Acteurs mobilisés: Les patient(e)s sont la catégorie la plus ciblée, suivis par les aidant(e)s, les citoyen(ne)s et les professionnels de santé. D’autres catégories sont également ressorties, comme les professionnels du champ social et de la prévention, des personnels associatifs	
	Critères de choix: Liés à la thématique de la recherche, majoritairement sur le critère de l’expérience d’une pathologie donnée	
	Moyens: Recrutement principalement grâce à un acteur relai et co-chercheur(e)s déjà impliqués dans un autre projet et par le «bouche à oreille»	

Les résultats révèlent que la majorité des chercheur(e)s répondants du réseau CANCEPT (10 sur 14) déclarent utiliser des démarches participatives dans leurs projets de recherche, avec des niveaux d’implication variables selon les étapes. Parmi les répondants, 8 sur 10 impliquent les personnes concernées dans la conception des projets, 5 sur 10 dans l’analyse des données, et seulement 1 sur 10 dans la préparation des appels à projets. Parmi les 4 chercheur(e)s n’ayant pas intégré de démarches participatives, les raisons invoquées incluent un manque de connaissance des méthodologies et une incompatibilité perçue avec la temporalité de leurs projets. Par ailleurs, certains chercheur(e)s ont indiqué pratiquer des formes de recherche participative, notamment en collaboration avec des associations, sans avoir conscience qu’il s’agissait d’une démarche participative formelle. Enfin, une réflexion a émergé autour de ce qui constitue une recherche participative, certaines approches consultatives ou unilatérales (comme les cohortes) étant jugées insuffisantes pour établir une recherche partenariale. Ces constats révèlent un besoin de clarification et de sensibilisation sur la recherche participative.

COPRICA, le groupe participatif de CANCEPT, a joué un rôle clé pour faciliter le recrutement dans la moitié des projets utilisant une approche participative. Il a permis aux chercheur(e)s d’identifier des personnes concernées dès les premières étapes, avant la soumission d’appel à projet. Cependant, un manque de connaissance sur le fonctionnement de COPRICA ou une inadéquation avec le contexte spécifique de certains projets a limité son utilisation. Pour pallier cette limitation, une démarche proactive a été initiée, comprenant des actions de sensibilisation et un accompagnement personnalisé des chercheur(e)s pour intégrer efficacement les démarches participatives et mobiliser COPRICA. Ces efforts visent à promouvoir des pratiques participatives robustes et durables au sein du réseau CANCEPT.

Parmi les principaux freins identifiés figure le manque de temps, les démarches participatives étant perçues comme chronophages, dû au besoin d’accompagnement pour légitimer et intégrer les partenaires dans leur rôle. À cela s’ajoutent un manque de financements pour valoriser leur participation, des difficultés à recruter des partenaires diversifiés, et un manque de connaissances méthodologiques et de formation, tant pour les chercheur(e)s que pour les co-chercheur(e)s. La méconnaissance des apports de la recherche participative et la difficulté à maintenir l’engagement des partenaires sur le long terme constituent également des obstacles majeurs.

Les leviers identifiés incluent une planification adaptée avec des délais et financements suffisants, l’intégration des co-chercheur(e)s dès le début et tout au long du projet, et un soutien méthodologique accru. L’accompagnement par COPRICA et l’accès à des exemples de projets participatifs réussis ont également été jugés essentiels pour encourager et structurer ces démarches. Une meilleure compréhension mutuelle des temporalités et des méthodes entre chercheur(e)s et co-chercheur(e)s est apparue comme une condition clé de réussite ainsi qu’un coordinateur pour créer une dynamique partenariale de groupe.

La plus-value de la recherche participative est ressortie comme évidente par ceux qui la pratiquent. Elle encourage une dynamique de groupe efficace, facilitant des échanges approfondis et une collecte de données plus précise. Les outils et messages de communication sont mieux adaptés, notamment en limitant leur caractère anxiogène. Les projets bénéficient d’une vision complémentaire qui les rend plus alignés avec les besoins des personnes concernées, améliorant ainsi leur efficacité. La participation des personnes concernées offre une perspective différente, ancrant la théorie dans la réalité du terrain. Comme l’ont souligné plusieurs chercheur(e)s, cette approche permet non seulement d’ajuster les projets, mais aussi de progresser en tant que chercheur(e)s grâce au contact direct avec les expériences vécues.

### Proposer des recommandations pour une implication efficace des personnes concernées en PP

#### Carte mentale pour illustrer les repères à l’implication des personnes concernées en recherche participative

À l'étape 3, le GEM MIXTE a cherché à surmonter les obstacles à l’implication des personnes concernées dans la recherche participative en PP. Bien que des lignes directrices existent, elles sont souvent sous-utilisées et n’intègrent pas toujours l’ensemble des personnes concernées au-delà des patient(e)s. Cette limite souligne la nécessité de développer des repères spécifiques pour adapter la recherche participative au contexte particulier de la PP.

**Fig. 2:**
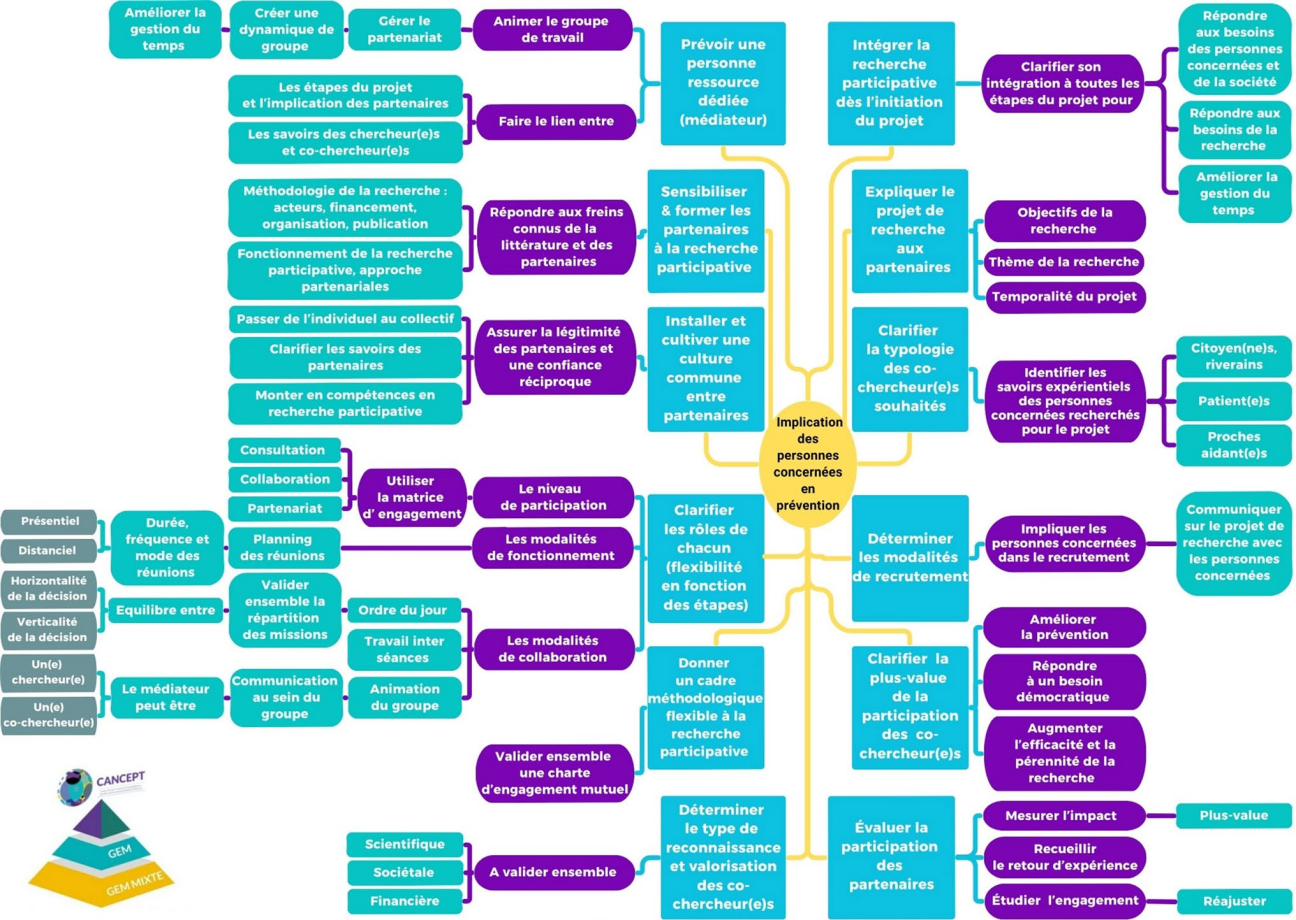
Carte mentale des repéres pour faciliter la recherche participative en pr**é**vention primaire

Nous avons choisi un format de carte mentale plutôt qu’une liste linéaire pour mettre en évidence l’ensemble des repères et pour considérer leur interconnexion, sans imposer de hiérarchie particulière. Présentée ci-après (Fig. 2), cette carte mentale constitue un outil structurant pour renforcer la recherche participative en PP.

Cette carte mentale apporte des réponses concrètes aux freins identifiés à l’étape 2, notamment en matière de gestion du temps, souvent perçue comme une contrainte majeure dans les projets participatifs. L’établissement d’un cadre méthodologique dès le début du projet peut aider à structurer les étapes et à réduire la charge de travail imprévue (Fig. 2).

Une charte d’engagement mutuel, élaborée entre chercheur(e)s et personnes concernées, constitue un outil essentiel pour définir les rôles et responsabilités de chacun. Cette charte offre également une flexibilité permettant d’adapter les contributions des membres en fonction des étapes du projet, favorisant ainsi une collaboration équilibrée et efficace.

La désignation d’une personne ressource pour animer le groupe de travail joue également un rôle clé dans l’optimisation du temps. Ce facilitateur peut garantir une bonne organisation, encourager la participation active et maintenir une dynamique productive au sein du groupe.

Enfin, la légitimation des personnes concernées dans un projet de recherche repose sur la reconnaissance de la complémentarité entre les savoirs scientifiques et expérientiels. Cette collaboration enrichit non seulement la qualité des travaux, mais elle constitue aussi un atout pour répondre plus efficacement aux besoins de la société et pour renforcer l’impact des actions de prévention. En associant ces savoirs, le partenariat en santé s’inscrit dans une démarche scientifique rigoureuse et inclusive, essentielle pour améliorer la recherche en prévention.

De nombreuses ressources ont alimenté cette carte mentale. Le guide co-construit avec des patient(e)s (Wilhelmy & CRCHUS, 2023) détaille les étapes clés de la recherche participative, incluant l'organisation du groupe, la formation, la légitimation des partenaires, et leur valorisation via compensations et reconnaissance scientifique. Le guide du NIHR (2022) est une référence pour initier des projets participatifs impliquant patient(e)s et public. Plusieurs articles récents ont mis en lumière des aspects clés de la recherche participative appliquée au domaine du cancer. Il s'agit notamment de l'importance d'une formation conjointe entre chercheur(e)s et patient(e)s partenaires afin de soutenir une compréhension commune et homogène des objectifs (Baillat et al., 2023), de la diversité des savoirs expérientiels des patient(e)s qui peut nourrir la recherche (Spears, 2021) et de l'allocation de fonds pour un chef de projet qui coordonne le groupe avec une triple fonction (socialisation, aspects pratiques et institutionnels). (Biaudet et al., 2024).

Ces constats corroborent les succès du groupe GEM MIXTE liés au temps de coordination dédié pour ce projet permettant non seulement la planification pratique des réunions (ordres du jour et comptes rendus) mais aussi la culture commune et le lien entre les différents acteurs du groupe et du projet sur le long terme.

L’évaluation de la participation des partenaires pour chaque projet de recherche participative – en prenant pour référence par exemple le PPEET (Abelson & Université McMaster, 2018)—permet d’en mesurer l’impact, de recueillir le retour d’expérience, d’étudier l’engagement des participant(e)s. Cette phase est importante pour déterminer d’éventuels réajustements et construire une recherche participative de plus en plus efficiente.

A l’issue de la conception de cette carte mentale, il apparaît que beaucoup de ces items sont communs à la recherche participative en santé. Néanmoins une particularité de la recherche partenariale en PP réside dans les enjeux de caractérisation des savoirs expérientiels mobilisés et de la posture des personnes concernées plus ou moins réflexive, vis-à-vis de l’objet de recherche. Ces savoirs expérientiels sont en lien avec des facteurs de risques liés à la santé ou à l’environnement et ne portent pas sur l’expérience d’une maladie.

Si ces recommandations dans notre carte mentale constituent des repères, il est important de les adapter au contexte avec les personnes concernées. Elles peuvent également évoluer en fonction du projet.

Chaque projet peut être co-construit, nécessitant une démarche participative continue, des espaces dédiés aux discussions, une évolution au fil du temps et laissant une large part à la souplesse, à la créativité tout en bénéficiant d’un cadre méthodologique scientifique.

#### Perspectives et limites du groupe GEM MIXTE

Le groupe GEM MIXTE, illustre l’intérêt et la faisabilité d’une recherche participative structurée dans le champ de la PP. Sa réussite repose sur plusieurs facteurs: l’élaboration d’une charte d’engagement mutuel pour clarifier les rôles et garantir une collaboration respectueuse, la coordination par une chercheure soucieuse d’assurer un équilibre dans les échanges, et une reconnaissance des co-chercheures au travers d’une indemnisation et d’une valorisation scientifique. Des formations dédiées ont permis de créer une culture commune, renforçant l’implication et la compréhension des concepts de recherche participative. L’intégration des co-chercheures dès la conception du projet et à toutes les étapes de celui-ci a favorisé leur engagement et leur appropriation des résultats. Il est parfois difficile de trouver l’équilibre entre verticalité et horizontalité des actions des co-chercheur(e)s, mais ce groupe a montré qu’ils définissent eux-mêmes les actions réalisables, comme partager des articles, travailler entre les réunions ou assumer le rôle de médiation à tour de rôle.

L’évaluation de la démarche participative au sein du GEM MIXTE à l’aide du questionnaire PPEET a révélé des résultats très positifs. Toutes les co-chercheures ont déclaré comprendre les objectifs du projet et disposer du soutien nécessaire pour leur participation, notamment via des compensations pratiques comme les défraiements. Elles ont unanimement estimé recevoir suffisamment d’informations pour jouer leur rôle, se sentir soutenues et libres d’exprimer leurs opinions, lesquelles sont systématiquement entendues et prises en compte. Elles ont également apprécié la diversité des perspectives partagées au sein du groupe et jugé que le projet atteint ses objectifs. Enfin, les participantes ont exprimé leur confiance envers les chercheur(e)s et ont souligné la plus-value du partenariat, affirmant que leur investissement avait un impact tangible et qu’il constituait un bon usage de leur temps tout en les enrichissant personnellement. Une des clés du succès mentionnées est le rôle dynamique de la coordinatrice du groupe de travail.

Cependant, des limites subsistent. Bien que des efforts importants aient été déployés pour recruter une diversité de personnes concernées, par le biais de diverses stratégies ciblées d’aller-vers, la majorité des co-chercheures étaient des patientes âgées de plus de 40 ans, issues du groupe COPRICA, ce qui a réduit la diversité des perspectives. Ces limitations dans le recrutement reflètent les défis rencontrés pour mobiliser une diversité de personnes dans un contexte où les patient(e)s, déjà sensibilisés aux enjeux de santé, se montrent généralement plus enclins à s’engager. C’est pourquoi nous avons co-construit un atelier participatif, qui s’est tenu lors du « 4e Colloque international sur le partenariat de soin avec les patient(e)s» à Lyon en 2024, dont les résultats serviront à rédiger un article sur la problématique du recrutement de co-chercheur(e)s en PP.

Pour surmonter ces limites, il est essentiel de diversifier le recrutement, de systématiser la mise en place d’une charte d’engagement et d’impliquer les participant(e)s dès les premières étapes des projets. Des formations appropriées ainsi qu'une évaluation régulière des démarches participatives peuvent améliorer l'efficacité et l'impact des actions mises en œuvre.

Ce travail issu du réseau CANCEPT s'inscrit dans une approche internationale. Les États-Unis, le Canada et le Royaume-Uni ont une solide expérience des recherches participatives intégrées dans les systèmes de santé et la recherche en santé (Cornish et al., 2023; Government of Canada, C. I. of H. R, 2014; Middel et al., 2024; Minkler et al., 2003; NIHR, 2022; *The PCORI Strategic Plan | PCORI*, 2025). Par exemple, le Royaume-Uni offre aux citoyen(ne)s la possibilité de s'engager dans la recherche en santé (https://bepartofresearch.nihr.ac.uk/). Dans le domaine de la prévention du cancer, plusieurs initiatives internationales illustrent comment la participation active des personnes concernées permet de mieux répondre aux déterminants sociaux de la santé, d’adapter les interventions aux contextes locaux et de renforcer leur durabilité (Bergin et al., 2023) En France, un éditorial récent (Olivier et al., 2024) appelle à un engagement accru en faveur de la recherche participative et à la normalisation de ces méthodes. Ce à quoi nous souhaitons contribuer en proposant cette carte mentale des recommandations en recherche participative en PP.

## Conclusion

Notre étude, éclairée par les contributions des personnes concernées, propose un cadre méthodologique pour faciliter la recherche participative en PP. L’implication des personnes concernées dotées de savoirs expérientiels variés permet de mieux comprendre les facteurs de risque et les comportements, contribuant ainsi à améliorer la recherche et à renforcer son impact sociétal. Nous avons identifié les obstacles, les leviers et des repères clés pour intégrer efficacement les personnes concernées dans ce domaine.

Bien que nécessitant un temps d’acculturation, la recherche participative augmente l’efficacité des projets en les alignant sur les besoins réels des communautés, justifiant ainsi l’investissement initial. La mobilisation d’acteurs relais pour le recrutement et l’utilisation de structures comme COPRICA allège la charge des chercheur(e)s tout en favorisant une implication précoce des personnes concernées En outre, cette démarche renforce les chances de financement en répondant aux priorités de démocratie sanitaire, et des réseaux comme CANCEPT jouent un rôle clé en sensibilisant les financeurs et en structurant les réponses aux appels à projets.

Ces repères offrent une base solide pour développer des interventions de PP plus adaptées et participatives, garantissant que les efforts de recherche soient représentatifs des communautés qu’ils visent à servir et maximisent leur impact. Nous espérons que ces repères inspireront d’autres réseaux pour structurer efficacement leurs démarches, renforçant ainsi la participation citoyenne en santé publique.

### Contributions à la connaissance

Qu’est-ce que cette étude ajoute aux connaissances existantes?

- Cette étude enrichit le champ de la recherche participative en PP, un domaine encore peu exploré. Elle met en avant:
- le rôle central d’un coordinateur pour animer la démarche participative,
- l’apport du savoir expérientiel des citoyen(ne)s pour adapter les interventions aux besoins réels,
- l’importance d’un groupe ressource comme COPRICA pour faciliter le recrutement et l’implication des partenaires.
- Réalisée au sein du réseau CANCEPT, elle propose un cadre méthodologique novateur combinant savoirs scientifiques et expérientiels, répondant ainsi aux besoins d’approches inclusives et durables en santé publique.

Quels en sont les principaux impacts, notamment en ce qui concerne les interventions, la pratique ou encore les politiques en santé publique?

- Cet article propose des recommandations méthodologiques pour structurer la recherche participative en PP. Le succès du GEM MIXTE illustre l’importance d’impliquer activement les personnes concernées, ce qui a été confirmé par leur forte adhésion aux objectifs du projet. L’état des lieux mené via un questionnaire co-construit au sein du réseau CANCEPT a permis de mettre en lumière les intérêts, les freins et les lacunes méthodologiques, tout en proposant des solutions concrètes. Ces résultats offrent des outils pratiques pour renforcer les démarches participatives, ouvrant la voie à des interventions de santé publique plus inclusives, durables et adaptées aux besoins des communautés.

## Data Availability

Non applicable.

## Disponibilité du code

Non applicable.

## Déclarations

### Approbation éthique

Non applicable.

### Consentement à participer

Nous avons obtenu le consentement éclairé de toutes les collègues de CANCEPT ayant participé au questionnaire.

### Consentement pour la publication

Non applicable.

### Conflit d'intérêts

Aucun conflit d’intérêts à déclarer.
